# P300/CBP Associated Factor (PCAF) Deficiency Enhances Diet-Induced Atherosclerosis in ApoE3^*^Leiden Mice via Systemic Inhibition of Regulatory T Cells

**DOI:** 10.3389/fcvm.2020.604821

**Published:** 2021-01-15

**Authors:** Alwin de Jong, Rob C. M. de Jong, Erna A. Peters, Ramon Arens, J. Wouter Jukema, Margreet R. de Vries, Paul H. A. Quax

**Affiliations:** ^1^Department of Surgery, Leiden University Medical Center, Leiden, Netherlands; ^2^Einthoven Laboratory for Experimental Vascular Medicine, Leiden University Medical Center, Leiden, Netherlands; ^3^Department of Hematology, Leiden University Medical Center, Leiden, Netherlands; ^4^Department of Immunology, Leiden University Medical Center, Leiden, Netherlands; ^5^Department of Cardiology, Leiden University Medical Center, Leiden, Netherlands

**Keywords:** cardiovascular disease, atherosclerosis, inflammation, PCAF, regulatory T cell

## Abstract

**Background:** Inflammatory stimuli induced by NF-kB drive atherosclerotic lesion formation. The epigenetic P300/CBP associated factor (PCAF) post-transcriptionally acetylates FoxP3, which is required for regulatory T-cell (Treg) differentiation and immune modulation. We hypothesize that PCAF deficiency affects atherosclerosis via regulation of regulatory Tregs.

**Method:** ApoE3^*^Leiden (*n* = 13) and ApoE3^*^LeidenxPCAF^−/−^ (*n* = 13) were fed a high-fat diet (HFD) containing 1.25% cholesterol. Systemic FoxP3^+^ T cells were measured every 4 weeks by flow cytometry (*n* = 6). After 5-months of HFD, mice were euthanized, and hearts and blood were collected. IL-6 and TNFα concentrations were measured in plasma to identify systemic inflammatory responses. Compositional and morphometrical analyses were performed on the atherosclerotic lesions in the aortic sinuses.

**Results:** After 5 months of HFD, plasma cholesterol concentrations were not different for ApoE3^*^LeidenxPCAF^−/−^ compared to ApoE3^*^Leiden mice. Expression of FoxP3 by systemic CD4^+^ T cells decreased 1.8 fold in ApoE3^*^LeidenxPCAF^−/−^ after 5 months HFD and remained significantly reduced after 5 months of HFD. Systemic TNFα and IL-6 concentrations were comparable, whereas the atherosclerotic lesion size in ApoE3^*^LeidenxPCAF^−/−^ mice was increased by 28% compared to ApoE3^*^Leiden mice. In atherosclerotic lesions, no differences were observed in macrophage differentiation or VSMC content, although a small increase in collagen was identified.

**Conclusion:** Our data show that PCAF deficiency resulted in a decrease in circulatory FoxP3^+^ regulatory T cells and ameliorated atherosclerotic lesions with no differences in systemic inflammation or macrophage differentiation in the atherosclerotic lesions. This suggests that PCAF regulates atherosclerosis via modulation of FoxP3^+^ regulatory T cell differentiation.

## Introduction

PCAF is known to acetylate histones, but also FoxP3. This acetylation prevents FoxP3 from proteasomal degradation in naïve T cells. Forkhead box P3 (FoxP3) drives the differentiation of regulatory T cells (Tregs) (1, 2). In human atherosclerotic lesions, the contribution of T cells is mainly via the CD4^+^ T cells and, to a lesser extent, the CD8^+^ effector T cells (3). The CD4^+^ T cells in these atherosclerotic lesions belong to the Th1 pro-inflammatory subset of helper T cells. In experimental atherosclerotic mouse models, T cells in atherosclerotic lesions show an antigen-experienced phenotype highlighting the inflammatory capacity of this effector T cells (4). The immunosuppressive Tregs modulate the activation of this effector T cell population. Naïve CD4^+^ T cells in the peripheral circulation differentiate Tregs, which is induced by the expression of the FoxP3 (5). Tregs were identified in atherosclerotic lesions and control the level of effector T cell activation and modulate the balance between the T_h_1 and T_h_2 response (6). Although Tregs have been found in relatively small amounts in atherosclerotic lesions, they significantly influence the stability of atherosclerotic lesions since even lower numbers were found in unstable atherosclerotic lesions (7).

It was demonstrated that the acetyltransferase PCAF is involved in cardiovascular diseases. Polymorphisms in the *PCAF* gene promotor were significantly associated with coronary heart disease mortality in elderly patients (8–10). In a post-interventional remodeling model, a deficiency in PCAF resulted in reduced tumor necrosis factor-α (TNFα) and interleukin (IL)-6 production in macrophages and subsequently reduced intimal hyperplasia *in vivo* (11). In PCAF deficient mice, impaired recruitment of leukocytes from the bone marrow was observed after induction of hind limb ischemia. A profound reduction of inflammatory effects, including a decrease in Tregs, resulted in the reduced collateral formation after induction of hind limb ischemia in these PCAF deficient mice (12).

The present study aimed to elucidate the effect of PCAF deficiency on atherosclerosis and specifically the role of PCAF on Treg in this process. Therefore, the expression of FoxP3 in T cells from hyperlipidemic ApoE3^*^Leiden mice vs. ApoE3^*^LeidenxPCAF^−/−^ mice and the contribution of PCAF to the atherosclerotic lesion size was studied.

## Materials and Methods

### Study Approval

This study was performed according to the Dutch government guidelines and the Directive 2010/63/EU of the European Parliament. The institutional committee of the Leiden University Medical Center approved all the animal experiments licensed under project number 11,076.

### Experimental Design

The generation of PCAF^−/−^ mice has been described previously, and Dr. C. Gongora kindly provided the mice (13). The PCAF^−/−^ were crossbred with ApoE3^*^Leiden, a strain in which a defective human ApoE gene is knocked in (14, 15). These mice still have their functional cholesterol metabolism and can be titrated with cholesterol-rich diets to specific plasma cholesterol levels. For the morphometric study, transgenic ApoE3^*^Leiden (*n* = 13) and ApoE3^*^LeidenxPCAF^−/−^ (*n* = 13) were fed with a high-fat high cholesterol type diet (HFD) containing 1.25% cholesterol to induce hypercholesterolemia (Ssniff). All animals receive food and water *ad libitum* during the entire experiment. Blood was taken from each mouse monthly for 5-months via tail puncture. After 5 months on HFD, the mice were anesthetized via an intraperitoneal injection of midazolam (5 mg/kg, Roche), medetomidine (0.5 mg/kg, Orion), and fentanyl (0.05 mg/kg, Janssen) followed by exsanguination via an orbital puncture. The thorax was opened and pressure-perfused (100 mm Hg) with phosphate-buffered saline (PBS, Braun) by a perforation of the left ventricle. After perfusion with 3.7% formaldehyde, the aortic sinus and heart were harvested and fixed in formalin for 24 h and subsequently embedded in paraffin. For the flow cytometry study, transgenic ApoE3^*^Leiden (*n* = 6) and ApoE3^*^LeidenxPCAF^−/−^ (*n* = 6) were fed with the HFD for 5 months. Blood was taken every month as described previously and processed for flow cytometric analysis. After 5 months of HFD, the mice were sacrificed as previously described and the blood, spleens and inguinal lymph nodes were isolated and processed for flow cytometric analysis.

### Blood Plasma Measurements

Total plasma cholesterol concentrations were measured enzymatically by using the Roche Diagnostic kit (Kit 1489437) according to the manufacturer's instructions. A serum triglyceride determination kit was used to measure triglycerides in mouse plasma samples (TR0100-1KT, Sigma Aldrich). Plasma concentrations of IL-6 and TNFα were determined by ELISA (BD, 555240, and 558534) and performed according to the manufacturer's instructions.

### Morphometric Analysis

The paraffin-embedded aortic sinuses were sectioned in 5 μm sections (Leica RM2355) and mounted on glass slides. After deparaffinization, the sections were stained with hematoxylin, phloxine, and saffron to determine the atherosclerotic lesion size. Atherosclerotic lesions containing predominantly layers of lipid-laden foam cells and mild fibrosis of the medial layer were classified as mild atherosclerotic lesions. Atherosclerotic lesions containing foam cells in the media, fibrosis, cholesterol clefts, mineralization, and necrosis of the media were classified as severe lesions (14). The necrotic core was identified as white areas in which the extracellular matrix was not present, within these areas cholesterol clefts and cellular debris can be observed. The atherosclerotic lesion area size was measured, and the largest three values averaged and represented as mm^2^ by using image analysis software (Caseviewer, 3D Histec).

### Compositional Analysis

The Sirius red staining visualized the collagen content, which was analyzed by FIJI (ImageJ). The Sirius red positive area is represented as the percentage of the total atherosclerotic lesion size. Anti-smooth muscle cell antibodies (1A4, 1:1,000, Dako) stained the vascular smooth muscle cells, visualized by the DAB substrate complex. Pro-inflammatory CCR2 expressing macrophages were stained with goat anti-mouse CD107b labeled Alexa Fluor 488 1:100 (Biolegend, M3/84) and rat anti-mouse CCR2 1:100 (Biolegend, SA203G11). Nuclei were visualized by Hoechst 34580 (1:1.000, Sigma, 63493) before mounting them with prolonged gold (Thermo Fischer, P36930). Images were obtained via laser scanning microscopy (LSM700, Zeiss). The counts of CD107b^+^ positive cells in the atherosclerotic lesions were calculated with FIJI. The CD107b^+^ and CCR2^+^ positive cells were counted in the atherosclerotic lesion area with FIJI.

### Flow Cytometric Analysis

The different tissues were minced and single-cell suspensions were obtained by using a 70 μm strainer, and the remaining erythrocytes were lysed for 1 min with red blood cell lysis buffer (A1049201, Thermo Fischer). Approximately 200 μl blood was taken monthly via a tail vein puncture. For flow cytometric analysis, the following antibodies were used: CD3 BV510 (1:100, Biolegend), CD4 PE-Cy7 (1:1,000, Thermo Fischer), CD8 BV605 (1:300, Biolegend), and FoxP3 BV421 (1:200, BD Biosciences), which incubated for 30 min at 4°*C*. The FoxP3 staining kit, including the fixation and permeabilization recommended by BD, was used according to the manufacturer's protocol (BD, 560409). For flow cytometric analysis, ~0.4 × 10^6^ cell fluorescent events were obtained by the BD Fortessa II analyzed with FlowJo VX.

### Statistical Analysis

Differences in continuous variables between experimental groups were statistically assessed by using the non-parametric *T*-test in Graph Pad Prism 8 software. Data are represented as means ± SD unless stated otherwise. Significance was set at *P* < 0.05. Significant differences are graphically represented as ^*^ *P* < 0.05, ^**^ *P* < 0.01, and ^***^ *P* < 0.001.

## Results

### Bodyweight, Cholesterol, and Triglyceride Levels Are Not Affected by PCAF Deficiency

**Figure 1:**
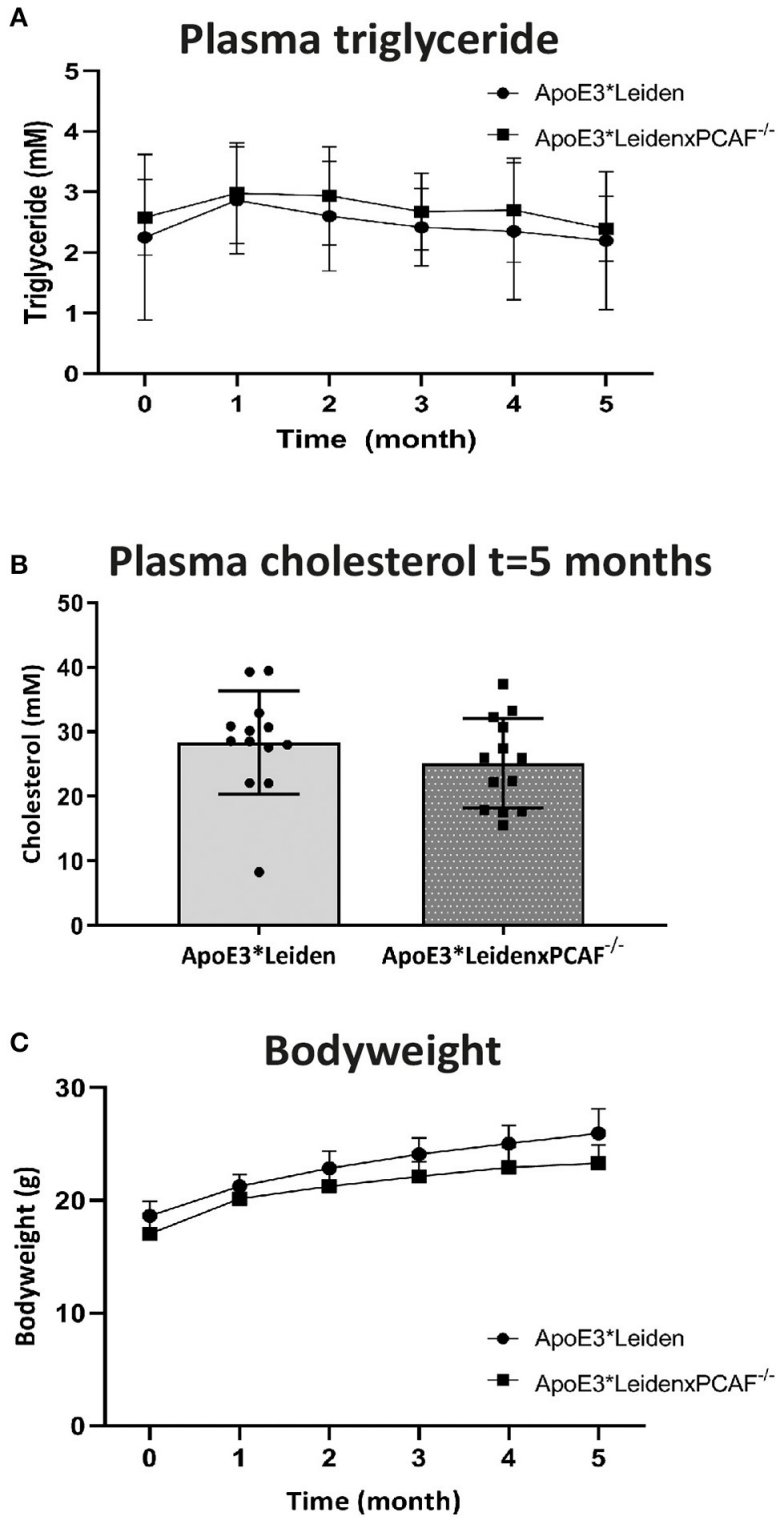
Bodyweight and plasma concentrations of triglyceride and cholesterol from ApoE3*Leiden and ApoE3*LeidenxPCAF^−/−^ mice after 5-months of the high-fat diet. Triglyceride concentrations **(A)** and total cholesterol **(B)** measured in plasma from ApoE3*Leiden (*n* = 13) and ApoE3*LeidenxPCAF^−/−^ (*n* = 13) fed with an HFD. Bodyweights of ApoE3*Leiden and ApoE3*LeidenxPCAF^−/−^ mice fed with an HFD **(C)**.

To examine the attribution of PCAF to atherosclerotic lesion development, we crossbred PCAF^−/−^ mice with atherosclerosis-prone ApoE3^*^Leiden mice. Female ApoE3^*^Leiden (*n* = 13) and female ApoE3^*^LeidenxPCAF^−/−^ transgenic mice (*n* = 13) were fed with an HFD continued for 5 months. Figure 1 shows the bodyweight plasma cholesterol and triglyceride concentrations of these HFD fed mice. The triglyceride concentrations have been determined at all time points and were found not to be different between ApoE3^*^Leiden mice and ApoE3^*^LeidenxPCAF^−/−^ mice (Figure 1A). The plasma cholesterol concentrations were not different between ApoE3^*^Leiden mice and ApoE3^*^LeidenxPCAF^−/−^ mice after 5 months on HFD (Figure 1B). ApoE3^*^LeidenxPCAF^−/−^ mice started with slightly lower body weight than ApoE3^*^Leiden mice, and this (non-significant) difference was sustained throughout the experiment (Figure 1C). Thus, a deficiency of PCAF did not affect body weight, plasma cholesterol, and triglyceride concentrations.

### ApoE3^*^LeidenxPCAF^−/−^ Mice Show Decreased Numbers of Circulating FoxP3^+^ T Cells

**Figure 2:**
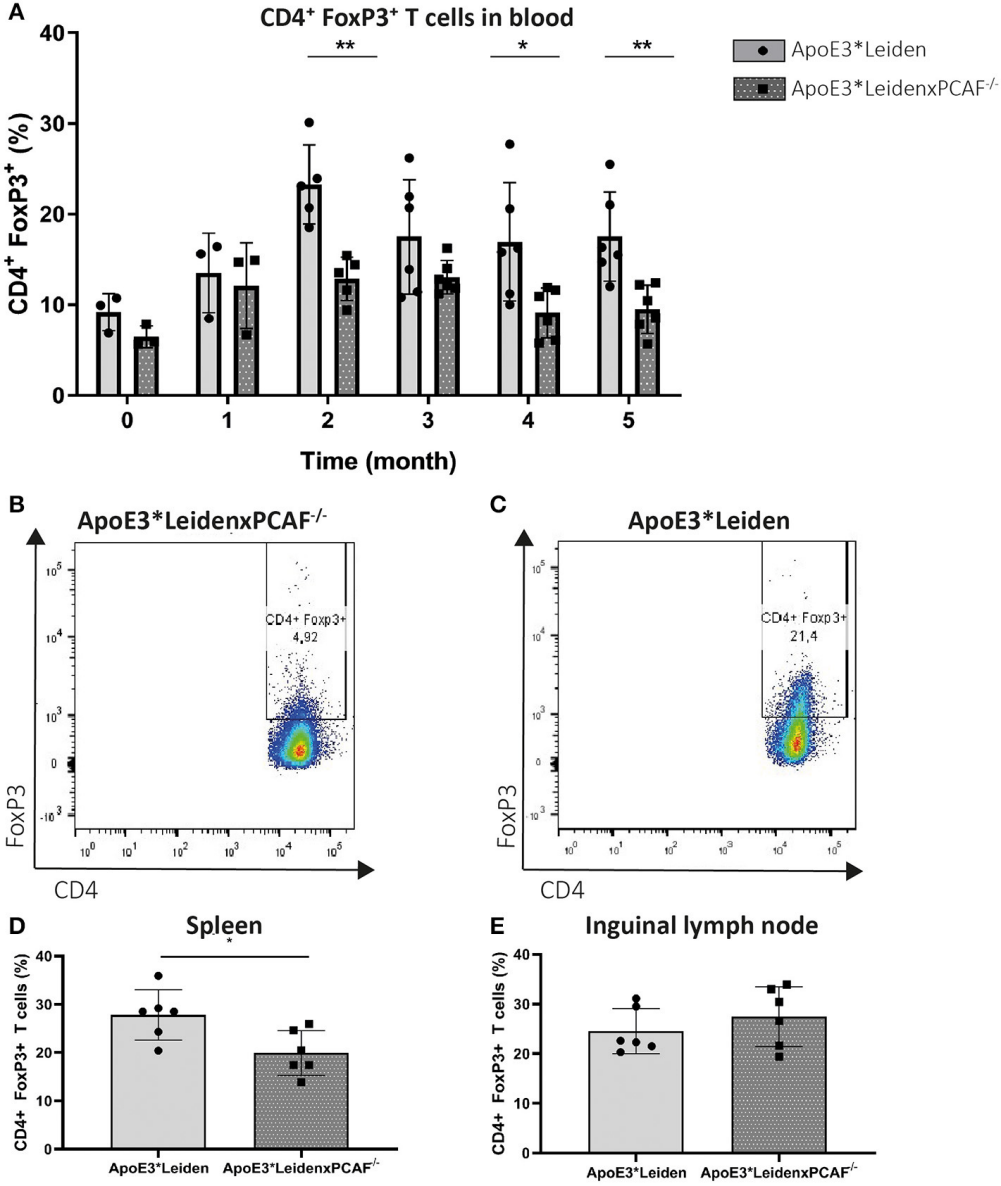
FoxP3 expression in time measured by flow cytometry in PBMC of ApoE3*Leiden and ApoE3*LeidenxPCAF^−/−^ mice. Peripheral blood mononuclear cells from ApoE3*Leiden and ApoE3*LeidenxPCAF^−/−^ mice (for both groups; *n* = 3 for timepoint 0 (before high-fat diet) and one month, *n* = 6 for the time points 2–5 months) were analyzed by flow cytometry. The percentage CD3^+^ CD4^+^ FoxP3^+^ cells in the PBMCs isolated from ApoE3*Leiden mice and ApoE3*LeidenxPCAF^−/−^ mice **(A)**. CD4^+^ FoxP3^+^ dot plot of PBMCs from an ApoE3*LeidenxPCAF^−/−^ mouse **(B)** and an CD4^+^ FoxP3^+^ dot plot of PBMCs from ApoE3*Leiden mouse **(C)** after 5-months of HFD. The percentage CD4^+^ FoxP3^+^ T cells in splenocytes **(D)** and inguinal lymph nodes **(E)** after 5-months of HFD. Non-parametric *T*-test *P < 0.05, **P < 0.01, and ***P < 0.001.

Next, we wanted to identify the contribution of PCAF Treg differentiation development. The percentage of FoxP3 expressing T cells in the peripheral blood mononuclear cells (PBMC) was evaluated every month by flow cytometry in 6 mice per group. PCAF deletion in ApoE3^*^Leiden mice did not affect circulatory T cell numbers compared to ApoE3^*^Leiden mice at the start of HFD or after 5 months of HFD (Supplementary Figure 1). The percentages of CD3^+^, CD4^+^, and FoxP3^+^ cells in PBMC are shown in Figure 2. After one month of HFD, the expression of FoxP3 was comparable between ApoE3^*^Leiden and ApoE3^*^LeidenxPCAF^−/−^ mice (Figure 2A). Throughout the HFD intervention, the percentage of FoxP3^+^ in the PBMCs of ApoE3^*^Leiden mice increased by the month. However, the differentiation rate of FoxP3^+^ cells in ApoE3^*^LeidenxPCAF^−/−^ mice showed a significant two-fold reduction compared to ApoE3^*^Leiden mice after 2 months of HFD (*p* = 0.004). The decrease in FoxP3 expression continued in ApoE3^*^LeidenxPCAF^−/−^ mice and remained significantly reduced after feeding the HFD for 5 months. However, this difference was not significant at the 3-months' time point (*p* = 0.13) (Figure 2A). Flow cytometric analysis showed that ApoE3^*^LeidenxPCAF^−/−^ mice (Figure 2B) had significantly lower percentages of FoxP3 expressing T cells in their peripheral circulation than ApoE3^*^Leiden mice (Figure 2C). Also, T cells isolated from the spleens of ApoE3^*^LeidenxPCAF^−/−^ mice showed a significant decrease in FoxP3^+^ T cells after 5 months of HFD (Figure 2D). However, this decrease in the percentage of FoxP3^+^ T cells was not observed in the inguinal lymph nodes (Figure 2E). A reduction in systemic FoxP3 expression suggests that PCAF contributes to the differentiation of Tregs present in PBMC.

### PCAF Deficiency Does Not Affect Systemic Pro-inflammatory Cytokine Levels

**Figure 3:**
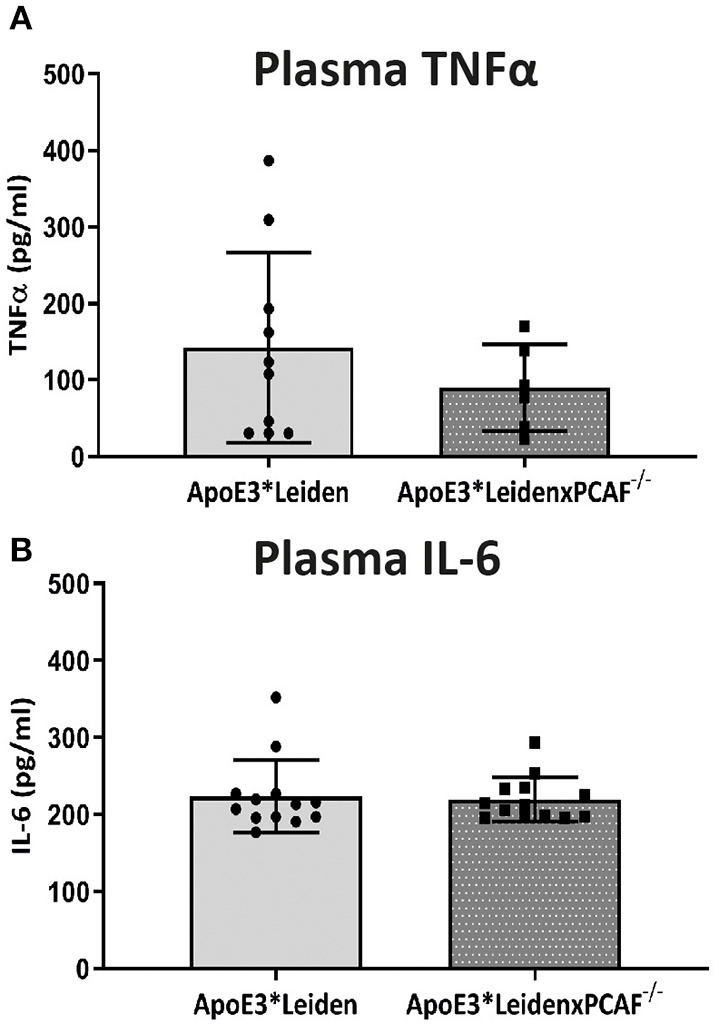
TNFα and IL-6 measurements in plasma. The concentrations of TNFα **(A)** and IL-6 **(B)** measured in plasma of ApoE3*Leiden mice (*n* = 13) and ApoE3*LeidenxPCAF^−/−^ mice (*n* = 13) after 5 months fed by an HFD.

To determine whether PCAF is required for pro-inflammatory cytokine expression, TNFα and IL-6 concentrations were determined in plasma of ApoE3^*^Leiden mice compared to ApoE3^*^LeidenxPCAF^−/−^ mice after five-moths of HFD. The plasma TNFα and IL-6 levels are represented in Figure 3. The TNFα concentrations in the plasma of ApoE3^*^LeidenxPCAF^−/−^ were reduced, but not significantly (*p* = 0.13). Besides TNFα, IL-6 is also a potent pro-inflammatory cytokine, but no differences were observed in the plasma IL-6 concentrations after five-moths of HFD (Figure 3B). This indicates that PCAF^−/−^, on a systemic level does not affect the TNFα or IL-6 plasma concentrations in transgenic ApoE3^*^Leiden mice.

### ApoE3^*^LeidenxPCAF^−/−^ Mice Show Increased Atherosclerotic Lesion Sizes

**Figure 4:**
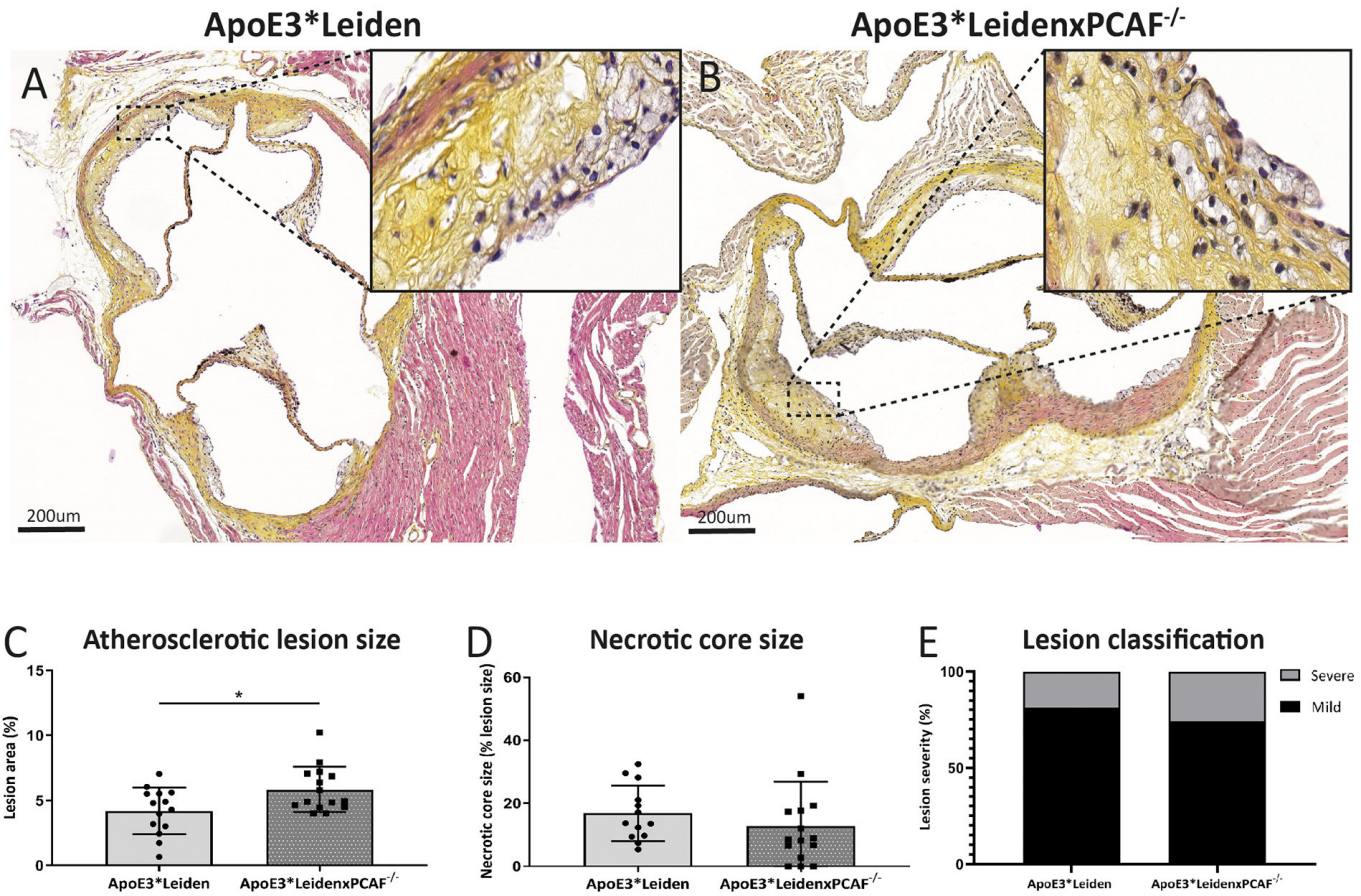
Morphometric analysis of atherosclerotic lesions in ApoE3*Leiden mice and ApoE3*LeidenxPCAF^−/−^ mice in diet-induced atherosclerosis. Representative cross-sections of a hematoxylin, phloxine, saffron stained aortic sinus from ApoE3*Leiden mouse **(A)** and ApoE3*LeidenxPCAF^−/−^ mouse **(B)**. The atherosclerotic lesion size in percentage **(C)**, percentage necrotic core size **(D)**, and the atherosclerotic lesion classification in ApoE3*Leiden (*n* = 13) and ApoE3*LeidenxPCAF^−/−^ mice (*n* = 13) **(E)**. Non-parametric *T*-test *p < 0.05.”

The contribution of PCAF on atherosclerotic lesion size was evaluated for the lesions present in the aortic sinuses. After 5 months of HFD, atherosclerotic lesions in the aortic sinuses were observed in ApoE3^*^Leiden mice represented in Figure 4A and from ApoE3^*^LeidenxPCAF^−/−^ mice in Figure 4B. These atherosclerotic lesions were characterized by intimal thickening, foam cell presence, calcifications, and necrotic cores. Morphometric analysis revealed a significant 28% increase in atherosclerotic lesion size in ApoE3^*^LeidenxPCAF^−/−^ mice (*n* = 13) compared to ApoE3^*^Leiden mice (*n* = 13) (p:0.02) (Figure 4C). No differences in the necrotic core size (Figure 4D) or atherosclerotic lesion classification (16) (Figure 4E) were observed.

### PCAF Deficiency Does Not Affect Macrophage Polarization in the Atherosclerotic Lesions

**Figure 5:**
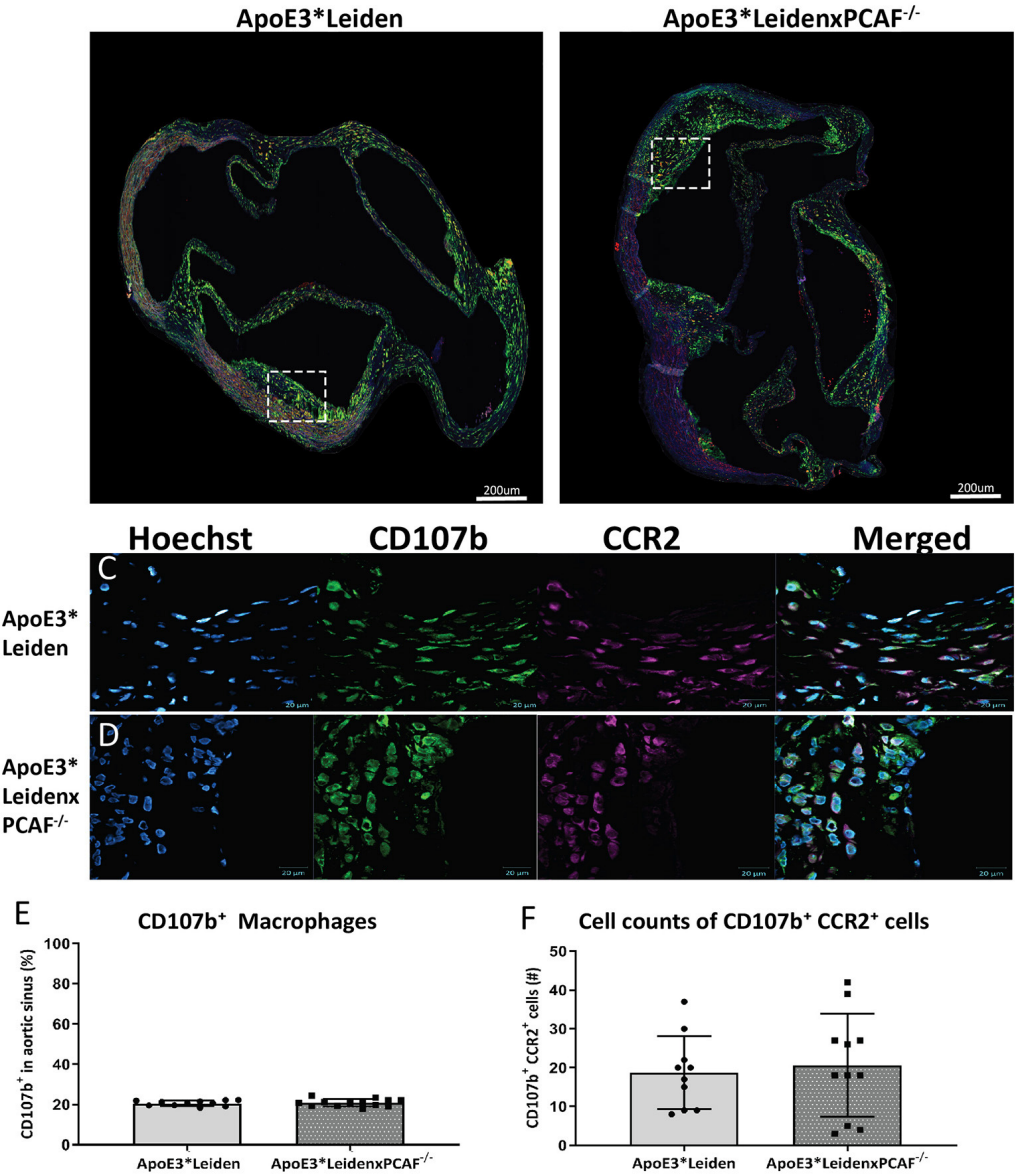
Measurements of CD107b expression in the total atherosclerotic lesion area and co-localization with CCR2. Representative images of the immunofluorescent stained aortic sinus**(A,B)**. At 40× magnification, a substantial overlap of the CCR2 signal with the CD107b was observed, which suggested that the macrophages present in the atherosclerotic lesions were pro-inflammatory macrophages **(C,D)**. No differences were observed in the area percentage of the CD107b expression in the atherosclerotic lesions located in the aortic of sinus ApoE3*Leiden and ApoE3*LeidenxPCAF^−/−^ mice **(E)**. Next, we analyzed the absolute cell count of the CD107b^+^ and CCR2^+^ cells **(F)**. This analysis showed no differences in the cell number of CD107b^+^ CCR2^+^ double-positive cells in the atherosclerotic lesions from ApoE3*Leiden compared to ApoE3*LeidenxPCAF^−/−^ mice.

The CD107b^+^ macrophage area and CCR2 expression in the atherosclerotic lesion were examined to determine if PCAF contributes to macrophage lesion content and if macrophages polarization toward a pro-inflammatory status in the atherosclerotic lesions. Representative images of the immunofluorescent stained aortic sinus are shown in Figures 5A,B. At 40× magnification, a substantial overlap of the CCR2 signal with the CD107b was observed, which suggested that the macrophages present in the atherosclerotic lesions were pro-inflammatory macrophages (Figures 5C,D). No differences were observed in the area percentage of the expression of CD107b in the atherosclerotic lesions located in the aortic of sinus ApoE3^*^Leiden and ApoE3^*^LeidenxPCAF^−/−^ mice (Figure 5E). Next, we analyzed the absolute cell count of the CD107b^+^ and CCR2^+^ cells (Figure 5F). This analysis showed no differences in the amount of CD107b^+^ or CCR2^+^ cells in the atherosclerotic lesions from ApoE3^*^Leiden compared to ApoE3^*^LeidenxPCAF^−/−^ mice.

### ApoE3^*^LeidenxPCAF^−/−^ Mice Show Increased Amounts of Newly Produced Collagen in the Atherosclerotic Lesions

**Figure 6:**
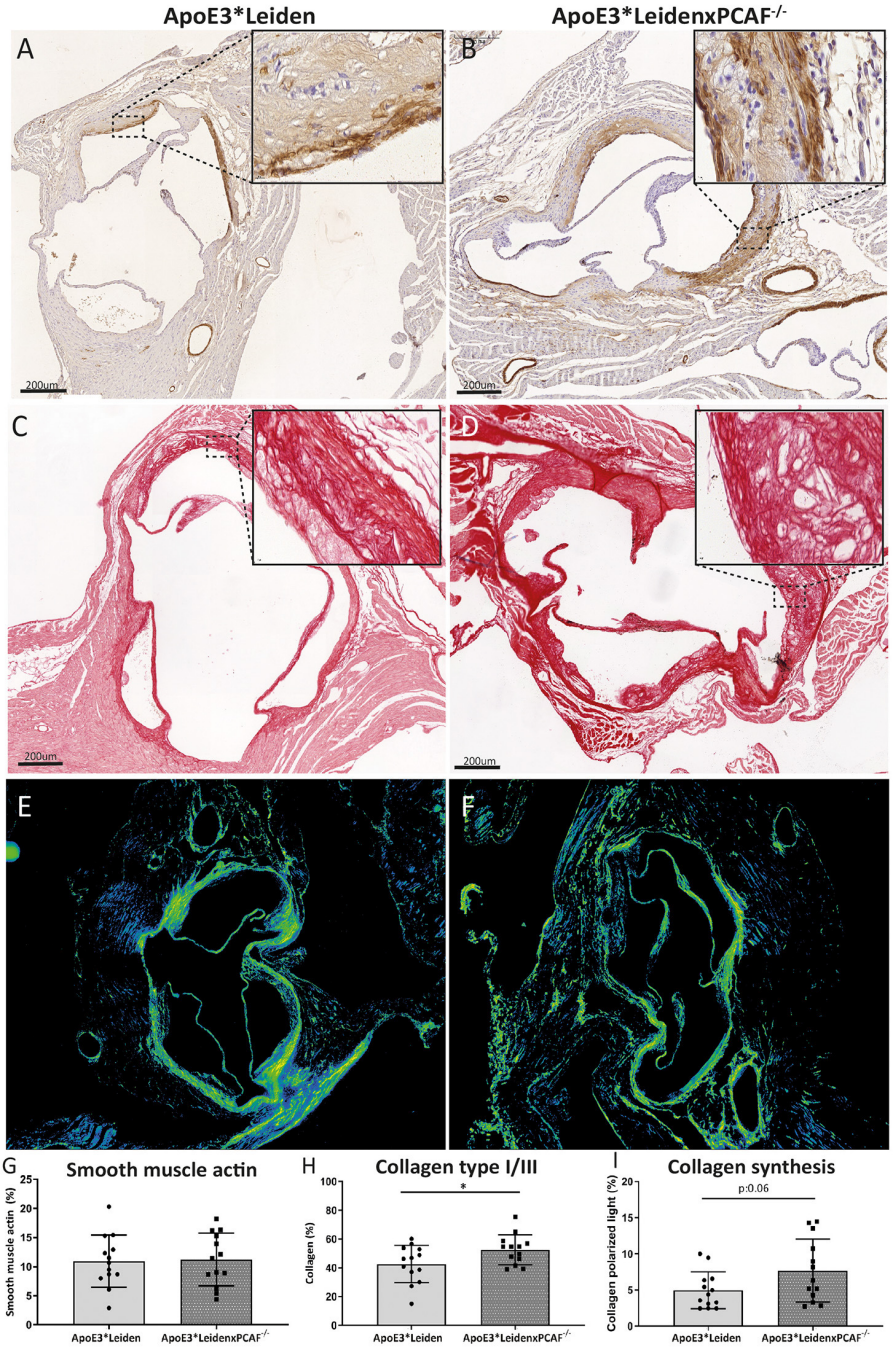
Vascular smooth muscle cells and collagen visualized in the atherosclerotic lesions. Representative images of smooth muscle cells in the aortic sinus of an ApoE3*Leiden mouse **(A)** and an ApoE3*LeidenxPCAF^−/−^ mouse **(B)** (bars 200 μm). Immunohistochemically stained αSMA can be observed on the luminal and medial side of the foam cells present in the atherosclerotic lesions. Representative images of collagen type I/III fibers were visualized in the aortic sinus of an ApoE3*Leiden mouse **(C)** and ApoE3*LeidenxPCAF^−/−^ mouse **(D)** (bars 200 μm). Newly formed collagen fibers were visualized by the use of polarized light microscopy **(E,F)**. Quantitative analysis of αSMA **(G)**, collagen **(H)**, and collagen under a polarized light condition **(I)** in the aortic sinus of ApoE3*Leiden mice (*n* = 13) and ApoE3*LeidenxPCAF^−/−^ mice (*n* = 13). Non-parametric *T*-test, *p < 0.05.

The VSMCs and collagen content was determined to examine the contribution of PCAF on the atherosclerotic lesion composition, which is represented in Figure 6. An overview of the VSMCs in an atherosclerotic lesion from an ApoE3^*^Leiden mouse depicted in Figure 6A and from an ApoE3^*^LeidenxPCAF^−/−^ mouse in Figure 6B. The percentage of VSMC in these atherosclerotic lesions was comparable between ApoE3^*^Leiden mice and ApoE3^*^LeidenxPCAF^−/−^ (Figure 6E). Collagen was visualized in the atherosclerotic lesion from ApoE3^*^Leiden mice (Figure 6C) and ApoE3^*^LeidenxPCAF^−/−^ mice (Figure 6D). The percentage of collagen present in the atherosclerotic lesions was slightly but significantly increased by 14% in ApoE3^*^LeidenxPCAF^−/−^ compared to ApoE3^*^Leiden mice (p=0.04) (Figure 6F). Next, we identified if this increase in collagen was due to a decrease in MMP9 presence. The expression of MMP9 was semi-quantitatively analyzed and showed no differences in the expression of MMP9 observed in the atherosclerotic lesions from ApoE3^*^Leiden compared to ApoE3^*^LeidenxPCAF^−/−^ mice (Supplementary Figure 2). To identify if the total collagen measured at 5 months of HFD was due to an increased production, polarized light microscopy was used to visualize newly produced collagen fibers (17). Analysis of these images showed a borderline significant increase in new collagen fibers in ApoE3^*^LeidenxPCAF^−/−^ mice compared to ApoE3^*^Leiden mice (Figure 6I).

## Discussion

Here we show that PCAF deficiency in ApoE3^*^Leiden mice is associated with a significant decrease in systemic FoxP3^+^ Tregs resulting in an increase in atherosclerotic lesion size in the aortic sinus. Although atherosclerotic lesion formation is inflammation-driven and PCAF is associated with inflammation (11), no differences in systemic TNFα or IL-6 levels were measured in plasma between the control and PCAF deficient mice. No differences in macrophage content or polarization were identified in atherosclerotic lesions from ApoE3^*^Leiden mice and ApoE3^*^LeidenxPCAF^−/−^ mice.

Previous studies described that a single nucleotide polymorphism in the promoter region of PCAF was identified in patients undergoing percutaneous coronary intervention (PCI), which determined the risk of developing clinical stenosis after PCI (18). In contrast to a model for PCI, PCAF deficient mice showed a reduced inflammatory response and a reduction in intimal hyperplasia (11). Furthermore, Garcinol, a PCAF inhibitor, attenuated inflammation and inhibited post-interventional accelerated atherosclerosis in ApoE3^*^Leiden mice (11, 19). Although experimental models of atherosclerosis and post-interventional stenosis share common processes such as the invasion of inflammatory cells, SMC migration, and extracellular matrix depositions, but the inflammatory component between the mouse models differs. Post interventional stenosis is characterized by a high-grade inflammation induced by vascular damage after vessel dilatation, whereas in atherosclerosis models, a continuous low-grade inflammation is observed. This is demonstrated by the high plasma TNFα concentration in the aforementioned femoral artery cuffed mouse model (11). In our diet-induced atherosclerotic model, the TNFα concentrations were approximately ten-fold lower, indicating a low-grade inflammatory response. The low-grade inflammation observed in the spontaneous atherosclerosis model used for our studies, with no significant differences in plasma levels of the pro-inflammatory cytokines TNFα and IL6 between the ApoE3^*^Leiden and ApoE3^*^LeidenxPCAF^−/−^ mice, may explain why PCAF deficiency did not result in smaller lesion size, as seen in the high inflammation grade post-interventional lesions, but rather in increased lesion size due to a reduction in Tregs in the ApoE3^*^LeidenxPCAF^−/−^ mice.

Our study showed the plasticity of FoxP3^+^ T cells by revealing an increase in FoxP3 expression over time in both the ApoE3^*^Leiden and ApoE3^*^LeidenxPCAF^−/−^ mice. Yamauchi et al. and Xiong et al. have shown that PCAF contributes to the induction of the *FOXP3* gene and stabilization of the FoxP3 protein (13, 20). Acetylating the lysine residues of FoxP3 is needed for the differentiation of naïve T cells into Tregs with immunosuppressive functions (13, 20). Bastiaansen et al. also showed a reduction in CD4^+^ T cells in PCAF deficient mice but, more importantly, a decrease in FoxP3^+^ Tregs (12). Depletion of peripheral Tregs by anti-CD25 monoclonal antibodies increased atherosclerotic lesion size and vulnerability in pro-atherogenic ApoE^−/−^ mice (21–24). Our PCAF deficient ApoE3^*^Leiden mice showed a reduced induction rate of Tregs over time. Loss of function of FoxP3 results in a lethal autoimmune response, while the deletion of either CBP or PCAF results in only a modest decrease in Tregs (25), as we also observed in ApoE3^*^LeidenxPCAF^−/−^ mice. Jia et al. showed that the loss of stable expression of FoxP3 in Tregs is associated with increased methylation of the *FOXP3* locus in patients with severe coronary artery disease (26). In human atherosclerosis, a reduction in Tregs was also associated with an increase in atherosclerotic lesion size as well as atherosclerotic lesion stability, and it negatively correlated with the presence of effector T cells (7). Others have shown that atherosclerotic disease is aggravated via a disbalance in Th17/Tregs, favoring the Th17 response (27). Tregs can promote the transformation of pro-inflammatory M1 macrophages to fibrotic M2 macrophages by releasing IL-10, which helps to prevent atherosclerosis (28). Here we observed that atherosclerotic lesions were primarily composed of pro-inflammatory macrophages. As shown by Marganto-Garcia et al., circulating Treg cell numbers in atherosclerosis in mice are reduced in later stages of the disease, whereas total CD4^+^ effector T cells and splenic Tregs increase with increasing atherosclerotic lesion size (29). These findings confirm our observations that a reduction in Tregs in PCAF deficient ApoE3^*^Leiden mice showed increased atherosclerotic lesion sizes.

Our data show that PCAF deficiency resulted in a decrease in circulatory FoxP3^+^ T cells. Although we did not observe differences in systemic inflammation or macrophage differentiation, PCAF deficiency led to an increase in atherosclerotic lesion size. This conclusion suggests that PCAF regulates atherosclerosis via modulation of FoxP3^+^ Treg differentiation.

## Data Availability Statement

The raw data supporting the conclusions of this article will be made available by the authors, without undue reservation.

## Ethics Statement

The animal study was reviewed and approved by The animal welfare institutional committee of the Leiden University Medical Center. Written informed consent was obtained from the owners for the participation of their animals in this study.

## Author Contributions

AJ, RJ, EP, and MV performed the experiments for the article. AJ, MV, JJ, and PQ wrote the manuscript. All authors contributed substantially to the discussion of content and reviewed and edited the manuscript before submission.

## Conflict of Interest

The authors declare that the research was conducted in the absence of any commercial or financial relationships that could be construed as a potential conflict of interest.

## Supplementary Material

Supplementary Figure 1 T cells at baseline and after 5 months of a high-fat diet. Peripheral blood mononuclear cells from ApoE3^*^Leiden and ApoE3^*^LeidenxPCAF^−/−^ mice (for both groups; *n* = 3 for timepoint 0 (before high-fat diet) and after 5 months of HFD. The CD3+ **(A,B)**, CD3+ CD4+ **(C,D)**, and CD3+ CD8+ **(E,F)** T-cells are represented.

Supplementary Figure 2 MMP9 staining on the aortic sinuses of ApoE3^*^Leiden and ApoE3^*^LeidenxPCAF^−/−^ mice. Representative images of the MMP9 immunohistochemical staining in the aortic sinus of an ApoE3^*^Leiden mouse **(A)** and an ApoE3^*^LeidenxPCAF^−/−^ mouse **(B)** (bars 200 μm). Semi-quantitative analysis of the MMP9 expression **(C)**.
