# Supplementary figures and images for: P300/CBP Associated Factor (PCAF) Deficiency Enhances Diet-Induced Atherosclerosis in ApoE3*Leiden Mice via Systemic Inhibition of Regulatory T Cells
Source: Front Cardiovasc Med. 2021 Jan 15;7:604821. doi: 10.3389/fcvm.2020.604821 (PMC7874080; doi:10.3389/fcvm.2020.604821)

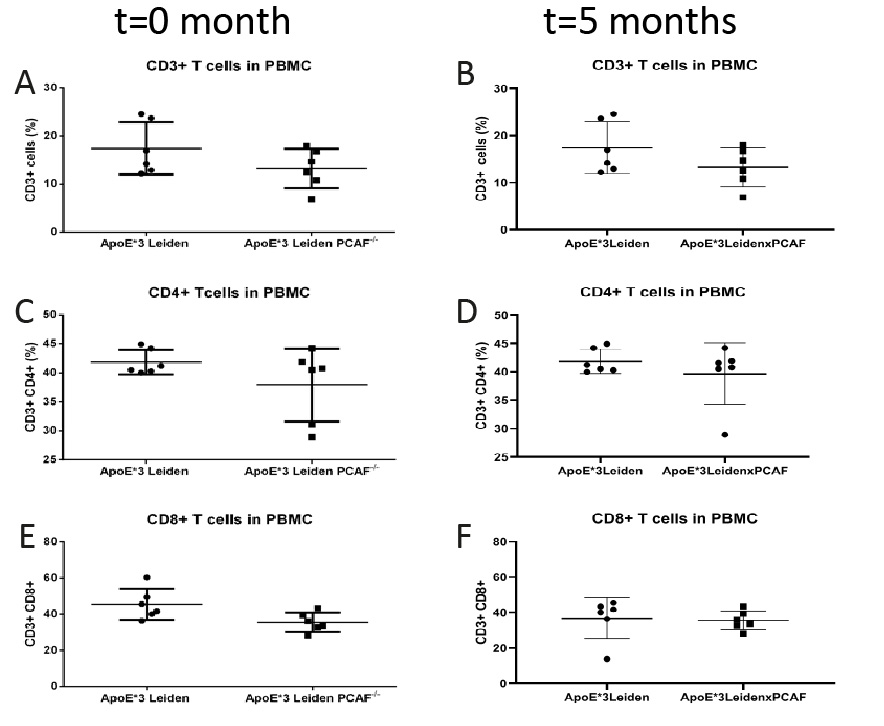

Supplement: Supplementary Figure 1 — T cells at baseline and after 5 months of a high-fat diet. Peripheral blood mononuclear cells from ApoE3*Leiden and ApoE3*LeidenxPCAF−/− mice (for both groups; n = 3 for timepoint 0 (before high-fat diet) and after 5 months of HFD. The CD3+ (A,B), CD3+ CD4+ (C,D), and CD3+ CD8+ (E,F) T-cells are represented. [file Image_1.JPEG]

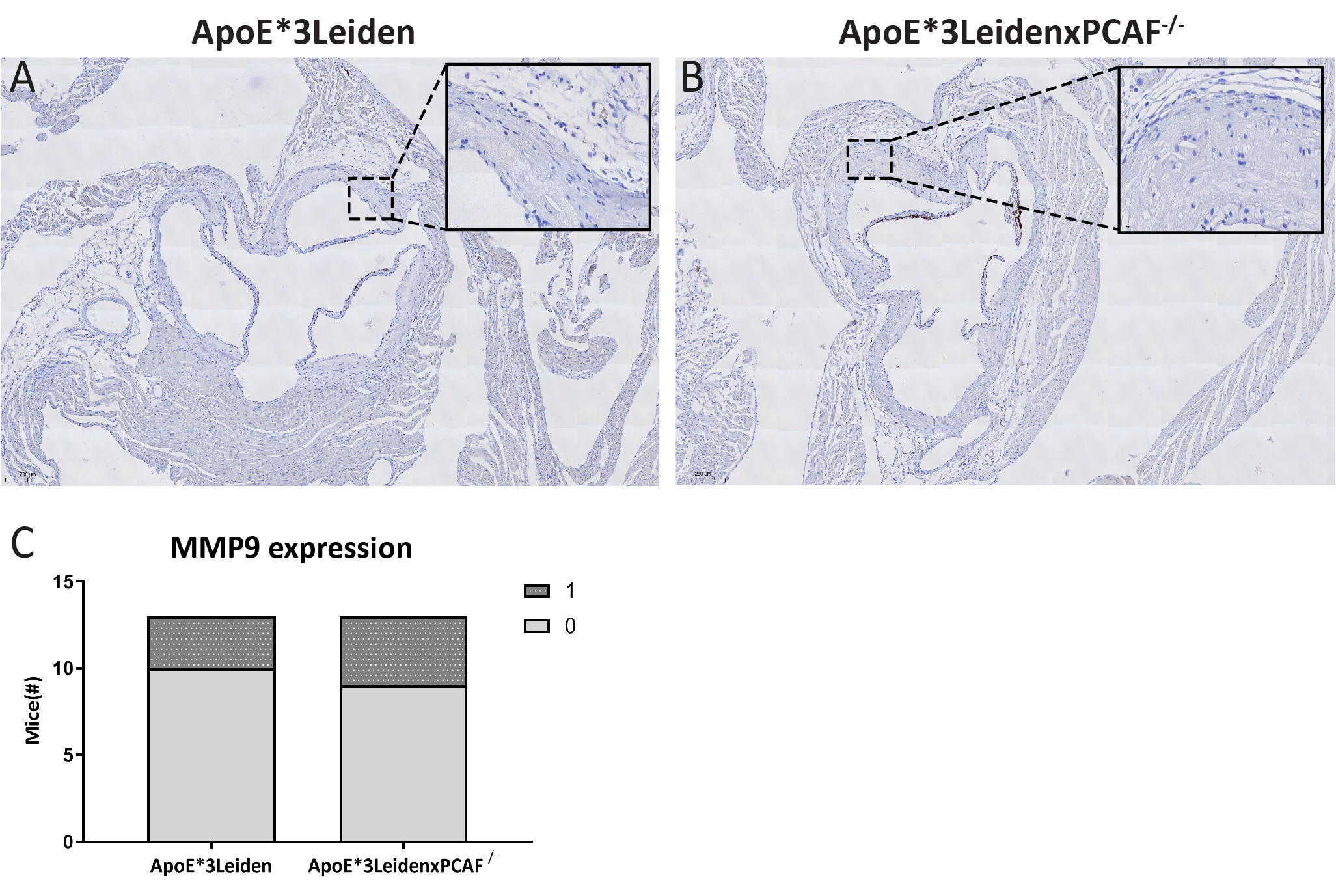

Supplement: Supplementary Figure 2 — MMP9 staining on the aortic sinuses of ApoE3*Leiden and ApoE3*LeidenxPCAF−/− mice. Representative images of the MMP9 immunohistochemical staining in the aortic sinus of an ApoE3*Leiden mouse (A) and an ApoE3*LeidenxPCAF−/− mouse (B) (bars 200 μm). Semi-quantitative analysis of the MMP9 expression (C). [file Image_2.JPEG]
